# The histological assessment of new bone formation with zolendronic acid loaded bone allograft in rabbit femoral bone defect

**DOI:** 10.25122/jml-2022-0314

**Published:** 2023-04

**Authors:** Dina Saginova, Elyarbek Tashmetov, Yevgeniy Kamyshanskiy, Amina Koshanova, Marietta Arutyunyan, Ibrahim Rustambek

**Affiliations:** 1 Center for Applied Scientific Research, National Scientific Center of Traumatology and Orthopaedics named after academician N.D.Batpenov, Nur-Sultan, Kazakhstan; 2 Department of Surgical Diseases, Karaganda Medical University, Karaganda, Kazakhstan; 3 Institute of Pathology of the University Clinic, Karaganda Medical University, Karaganda, Kazakhstan

**Keywords:** bone allograft, bone regeneration, zolendronic acid, Marburg bone bank system

## Abstract

The aim of this experimental study was to evaluate the effect of zolendronic acid (ZOL) combined with bone allograft prepared using the Marburg Bone Bank System on bone formation in the implant remodeling zone. Femoral bone defects with a diameter of 5 mm and a depth of 10 mm were created in 32 rabbits. Animals were divided into 2 similar groups: Group 1 (control), where defects were filled with bone allograft, and Group 2, where allograft was combined with ZOL. Eight animals from each group were sacrificed at 14- and 60-days post-surgery and bone defect healing was assessed using histopathological and histomorphometric analyses after 14 and 60 days. The results showed that new bone formation within the bone allograft was significantly greater in the control group than in the ZOL-treated group after 14 and 60 days (p<0.05). In conclusion, local co-administration of ZOL on heat-treated allograft inhibits allograft resorption and new bone formation in the bone defect.

## INTRODUCTION

Bone substitutes are widely used in the surgical treatment of musculoskeletal pathology [1-3], especially in revision arthroplasty of large joints [2,4]. In these cases, bone implants not only provide adequate time for the formation of biological stability but also preserve the primary stability of the endoprosthesis during stress remodeling [4]. However, in revision arthroplasty, the acceleration of graft resorption during revascularization is not always accompanied by adequate bone formation, which reduces the strength properties of the graft and poses a threat of aseptic instability of the endoprosthesis in the early period after surgery [1,4-7]. The risk of instability increases in cases where there are preoperative disorders of bone remodeling (endoprosthesis against the background of systemic osteoporosis or aseptic necrosis of the femoral head) [8].

Local application of bisphosphonates, a group of synthetic pyrophosphates, has been shown to reduce the intensity of implant resorption and preserve its osteoconductive effect, creating conditions for the formation of biological stability by normalizing remodeling [9,10]. However, there are differing opinions among specialists regarding the use of bisphosphonates locally [11,12].

Despite the wide use of bisphosphonates as systemic antiresorptive drugs for treating systemic osteoporosis, early prevention of aseptic instability of endoprostheses, aseptic necrosis, and metastatic lesions of bones, their local application in surgical interventions, including in biomaterials, remains a topic of debate [5,11]. The problem of "retention" of bisphosphonates in the surgical intervention zone due to their inability to persist as a solution has not been resolved [9]. Various materials, such as collagen, demineralized bone matrix, and polymers of natural and synthetic origin based on polyglycolic and lactic acids, have been proposed as special bioinert carriers [13-16]. However, the use of these carriers only confirms the ongoing unsolved problem. The purpose of this study was to evaluate the effect of zolendronic acid with bone allograft prepared according to the Marburg Bone Bank System on the process of bone formation in the bone implant remodeling zone.

## Material and Methods

### Preparation of bone allografts

In this study, a bone allograft from the head of the femur from a living donor (after arthroplasty) was used in accordance with the national legislation of Kazakhstan [17,18].

The bone transplant, namely the head of the femur, was obtained from patients who underwent arthroplastic surgery (hip joint endoprosthesis). The femoral head was taken in the operating room and subjected to mechanical cleaning of soft tissues, cartilage, and ligaments under sterile conditions. Afterward, the femoral head was placed in a disposable sterile disinfection container and poured with a NaCl solution of 0.9% - 300 ml. The container was then closed and placed in the Lobator heat treatment device. The processing cycle was 94 minutes [19]. At the end of the cycle, through a special hole in the container, a fence for sterility was made, then the liquid (broth) was completely drained. The bone allograft was then stored in a freezer at a temperature of -80°C following the protocol. Two hours before the experiment, the femoral head was unfrozen at room temperature and cut into chips.

### Preparation of zolendronic acid

Zolendronic acid (Sun Pharmaceutical Industries Ltd, India) at a concentration of 0.05 mg/ml (100 µl) [20] was added to 0.5 g of bone chips by soaking and kept in a sterile container.

### Animals and surgical procedures

**Figure 1:**
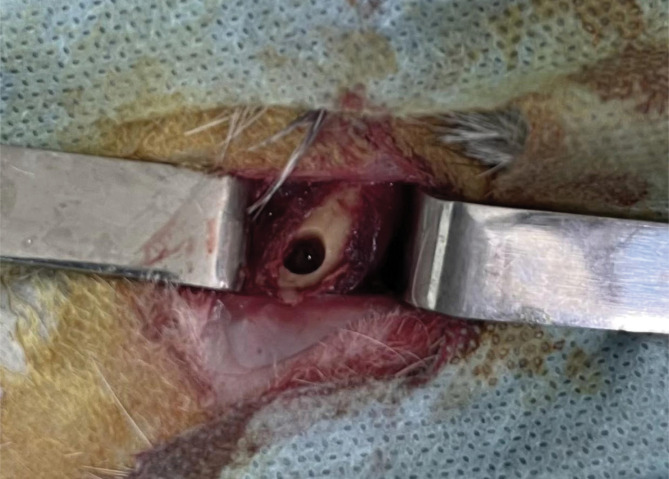
Femoral bone defect.

The study was conducted on 32 adult rabbits weighing 2530 ± 83 g, acclimatized for 14 days before the experiment. The rabbits were housed in special cages at a temperature of 22 ± 2 °C, with a humidity of 40%–50% and a 12-hour light-dark cycle. The study was conducted in accordance with the European Convention for the Protection of Vertebrate Animals used for Experimental and Other Scientific Purposes [21]. All animals were randomly divided into 2 equal groups of 16 rabbits each. Three hours before surgery, rabbits received an intramuscular (i.m.) injection of gentamicin 0.1 ml/kg (MAPICHEM, Switzerland). Under anesthesia with Zoletil 0.1 mg/kg (Virbac, USA) and Rometar 5 mg/kg intramuscularly (Bioveta, Czech Republic), a bone defect was formed on the distal metaphysis of the femur with a diameter of 5 mm and a depth of 10 mm using a bur machine (Figure 1).

**Figure 2:**
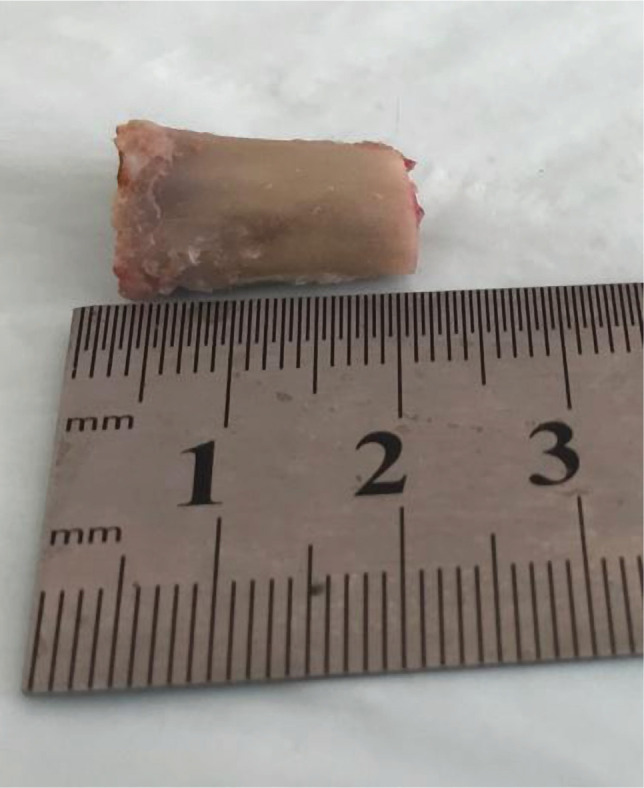
A fragment of the distal metaphysis of the femur in Group 1 with a healed defect after 60 days

In the first group, bone tissue defects were filled with a bone allograft (AG group). In the second group, the defects were filled with bone allografts with 5 µg zolendronic acid (AG+Zol group) (Sun Pharmaceutical Industries Ltd, India). The surgical wound was sutured with Vicryl 5.0 (Ethicon, Johnson & Johnson, USA). After the operation, each animal received intramuscular injections of gentamicin 0.1 ml/kg (MAPICHEM, Switzerland) and ketonal 0.04 ml/kg (Sandoz, Slovenia) for 3 days. The postoperative observation was carried out daily to assess the vital activity of rabbits. At 14- and 60-days, animals were sacrificed by an overdose of Zoletil 50 mg/ml, and the distal femur was harvested (Figure 2).

### Histological examination

The object for histopathological examination was a bone fragment with a formed defect. Before histological evaluation, the bone fragments were fixed in 10% neutral buffered formalin for 24 hours and decalcified in Biodec R solution (Bio-Optica Milano SPA) for 24 hours, after which the samples were washed in phosphate buffer (pH = 7.4). After optimal softening of the bone tissue (decalcification), a bone incision was performed. The tissue was fixed in 10% formalin at 4 ° C for 24 hours, washed with tap water, and dehydrated with a series of increasing alcohol concentrations (70%, 90%, 95%, 100%), then immersed in xylene and embedded in paraffin blocks. Tissue sections with a thickness of 5 µm were made on a Leica SM 2000R sled microtome. After preparation, the tissue sections were stained using hematoxylin and eosin staining (to determine the general tissue morphology and the cellular composition of the bone defect) and Masson’s trichrome staining (to determine the percentage of fibrous tissue, cartilage tissue, and bone tissue in the bone defect area). Microscopic examination of the preparations was conducted on a Zeiss AxioLab 4.0 microscope with magnifications of x200 and x400. AxioVision 7.2 software for Windows was used to analyze and photograph the images. Calculation of the cellular composition in the bone defect area (osteoclasts, osteoblasts, and osteocytes) was carried out in sections stained with hematoxylin and eosin: the number of osteoclasts, osteoblasts, and osteocytes was counted per 1000 cells on the bone defect, and the obtained mean values were expressed with an accuracy of 2 decimal places for each group.

### Statistical analysis

We conducted the statistical analysis using IBM SPSS statistical software. We expressed all experimental data as mean values and standard deviations (SD). We assessed differences between the two groups using the Chi-square test with Yate's correction and the Mann-Whitney test. For multiple comparisons, we used Pearson's Chi-square test. We considered a level of significance at P < 0.05.

## Results

**Figure 3:**
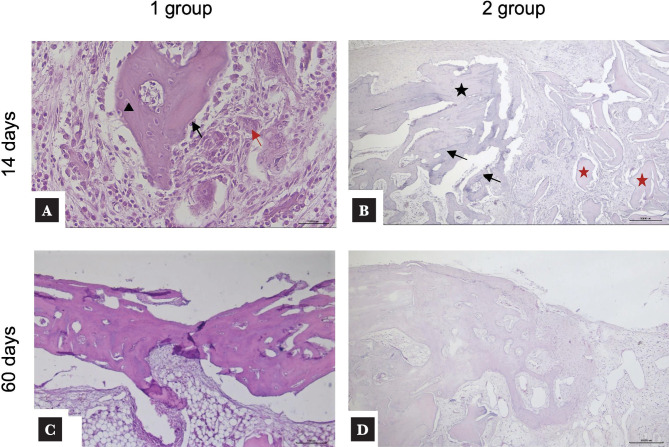
Histological sections 14 and 60 days after implantation: A – osteoblasts (black arrow) and giant multinucleated osteoclasts (red arrow) are observed on the bone surface; also, osteocytes (arrowhead) surrounded by a bone matrix); B – the area of the defect was partially closed by fibrous tissue and minimally resorbed allograft fragments (red star). Forming fibrous tissue with single thin-walled vessels and scanty infiltrate. Along the edge of the bone plate (black star), single chaotically located bone trabeculae (black arrows) are defined C – bone is presented in the form of chaotically located bone beams and cords to organize lamellar structures; D – the defect area is closed by fibrous tissue without cellular infiltration. In the bone defect edge, chaotically fragments of newly formed bone spreading into the intramedullary space are detected, and osteocytes and osteoblasts lining the newly formed bone are seen. (HE × 400; scale bar, 50000 um).

The histological image of the cellular composition of the bone plate defect after 14 days showed that the average number of osteoblasts in Group 1 was 296 ± 25.4 cells, while the numbers of osteoclast and osteocyte were 10.0 ± 7.4 cells and 212.5 ± 24.7 cells per 1000 cells per the area of the defect zone, respectively (Table 1) (Figure 3 A-D). In Group 2, the mean number of osteoblasts was significantly lower than in Group 1, but osteocyte numbers were higher. There was no significant difference in osteoclast numbers between Groups 1 and 2 (p>0.05) (Table 1). At day 60, osteoblast and osteocyte numbers in Group 1 were significantly higher than in Group 2 (p>0.05). However, no significant difference was observed between Groups 1 and 2 with respect to osteoclast numbers (p>0.05).

**Table 1:** Histomorphometric characteristics of the cellular composition of the bone defect.

No	Indicator name *	Group 1	Group 2	P-value
**14 days**				
**1**	Osteoblasts	296±25.4	255.2±16.2	<0.051
**2**	Osteoclasts	10.0±7.4	10.8±3.4	>0.753
**3**	Osteocytes	212.5±24.7	266.7±29.7	<0.044
**60 days**				
**1**	Osteoblasts	387±52.6	335.0±37.9	<0.026
**2**	Osteoclasts	6.67±4.3	5.9±3.2	>0.832
**3**	Osteocytes	394.6±40.7	344.9±28.6	<0.012

* Per 1000 cells in the defect zone. Data are presented as mean±standard deviation.

After 14 days, all animals in Group 1 exhibited new bone formation in the bone defect area, with an average bone tissue area of 43.3 ± 6.5 (Table 2). The newly formed bone was viable with lacunae containing osteocytes and vascular channels. Histologically, the trabecular meshwork of the newly formed bone was connected to the bone of the perforated allograft. The bone tracts of newly formed bone tissue were heterogeneous, predominantly thin, with focal bridge-like areas and single contacts, mainly at the poles of the bone tracts. Multiple vascular bundles were observed in the area of bone defect in both groups.

**Table 2:** Histomorphometric characteristics of the tissue composition of the bone defect.

No	Indicator name, %	Group 1	Group 2	P-value
**14 days**				
**1**	Fibrous tissue	45.1±4.4	52.5±3.8	<0.029
**2**	Cartilage tissue	11.6±1.2	17.7±4.3	<0.001
**3**	Bone	43.3±6.5	29.8±8.6	<0.011
**60 days**				
**1**	Fibrous tissue	2.2±1,2	52.3±6.1	<0.000
**2**	Cartilage tissue	7.5±4.7	10.7±5.3	<0.017
**3**	Bone	90.3±7.4	41.8±3.8	<0.000

Data are presented as the mean value±standard deviation

**Figure 4:**
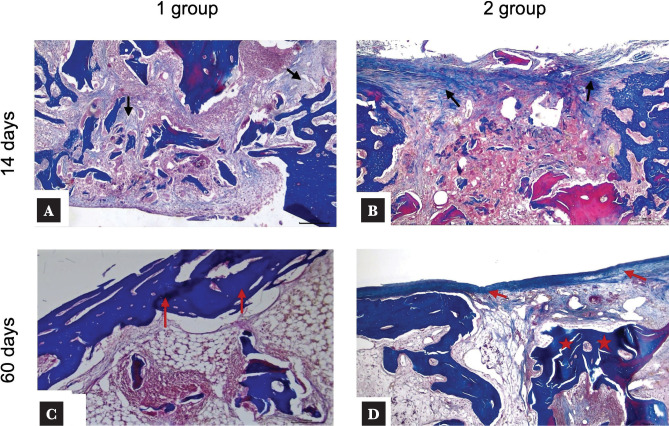
Histological sections 14 and 60 days after implantation: A, B – the fibrous layer surrounds (black arrow) the surface of the remodeling allograft with newly formed bone tissue; C – remodeling of newly formed bone tissue in the bone defect area (red arrow); D – the defect area is closed by fibrous tissue (red arrow) and allograft fragments (red star) with minimal resorption and remineralization. The forming bone tissue spreads perpendicular to the cortical plate into the intramedullary space. (Masson's trichrome × 200; scale bar, 50000 um).

The average area of fibrous tissue on day 14 in Group 1 was 45.1 ± 4.4%, while the cartilage tissue was 11.6 ± 1.2% (Table 2). In Group 2, the defect area was closed mainly by fibrous tissue 52.5± 3.8% with minimal cellular infiltrate, and there were allograft fragments with minimal resorption, integrating with the forming bone trabeculae (Figure 4). The fibrous tissue was represented by the focal formation of fibrous connective tissue fibers, predominantly at the periphery of the bone allograft, without spreading beyond the representative area of the bone defect (Figure 4 A, B). The average bone and cartilaginous tissue area were 29.8± 8.6% and 17.7± 4.3% of the area, respectively (Table 2).

On day 60, the bone tissue in Group 1 was characterized by osteocytes in the lacunae of newly formed bone and a dense basophilic line, indicating a high degree of mineralization and active longitudinal growth (Figure 4 C). The average area of bone tissue was 90.3±7.4%, while fibrous and cartilage tissue accounted for 7.5±4.7% and 2.2±1,2%, respectively (Table 2). In Group 2, the defect area was closed by fibrous tissue at 52.3±6.1% and bone at 41.8±3.8% (Figure 4 D). Thin and chaotically arranged newly formed bone trabeculae extended from the edge of the bone plate into the intramedullary space, with a clearly visible boundary between the bone plate and the newly formed bone. The newly formed bone tissue mainly consisted of thin and chaotically arranged bone trabeculae that extended into the intramedullary space, surrounded by a fibrous layer without any reactive infiltration at the surface of the newly formed bone beams and the edge of the cortical plate defect (Table 2).

## Discussion

Bisphosphonates have been shown to have a local effect when administered systemically in treating Paget's disease [21], as well as in patients at risk of early aseptic instability of endoprosthesis after joint arthroplasty [11]. However, the appropriateness of their local application in combination with bone implants [16] for the surgical treatment of musculoskeletal pathology [1], especially revision arthroplasty [22], remains a subject of debate.

According to some researchers [22], combining a graft with bisphosphonate reduces the intensity of resorption and thus not only preserves the mechanical strength of the graft but also prolongs its osteoconductive effect. The effectiveness of the local application of bisphosphonates in revision arthroplasty was confirmed in a randomized study by Kesteris and Aspenberg [23]. However, more recent experimental work [15] revealed that the use of bisphosphonates reduces bone formation and leads to a violation of the strength of implant fixation. The noted in vitro ability of bisphosphonates to reduce the intensity of resorption and bone formation [24] raises concerns among some researchers about their local application.

In light of the above, it seemed relevant to conduct a comparative assessment of the intensity of bone formation in bone allograft placement, prepared according to the Marburg system, containing the bisphosphonate zolendronic acid, using an appropriate control. This is especially relevant given the increasing use of zolendronic acid not only in treating systemic osteoporosis but also in managing bone fractures, complicating its course [25,26].

When comparing the intensity of bone formation in the bone defect area combined with the use of bisphosphonates, the effectiveness of bone formation activation was significantly higher in the control group, which used bone allograft without bisphosphonates on days 14 and 60. This result confirms the outcomes of earlier studies regarding the topical application of zolendronic acid in combination with bone biomaterials. In vitro studies have shown that bisphosphonates reduce the viability of osteoblasts in a dose-dependent manner.

In a study on dogs, Jakobsen et al. [20] impregnated lyophilized allografts with zolendronic acid at concentrations of 0.005 mg/ml, 0.05 mg/ml, and 0.5 mg/ml. The study found that the concentration of zolendronic acid significantly affected the amount of newly formed bone tissue around the allograft, with the highest amount of new bone formation observed in the group with the lowest concentration and no new bone formation observed in the group with the highest concentration. In the present study, the histological analysis showed that using zolendronic acid at a concentration of 5 µg per 0.5 g of bone allograft resulted in a lower area of newly formed bone (Table 1) and a decreased number of osteogenic cells (Table 2) at all stages of the study compared to the control group. This finding supports the idea, as noted by other authors [27,28] that the toxic effect of a high dose of bisphosphonate can block bone metabolism, osseointegration, and the bioimplant.

We found a negative effect of bisphosphonate on bone resorption compared to the control group. In the zolendronic acid-free group, the defects were replaced by newly formed bone, and the allograft was resorbed and replaced by fat-rich marrow in the reconstructed portion of the graft (Figure 4 C). However, in the zolendronic acid group, there was a large amount of preserved allogenic bone in the bone defect (Figure 3 D), indicating a greater inhibitory effect of topical administration of zolendronic acid. Bone allografts have osteoconductive and osteoinductive properties. During allograft resorption, BMP-2 and other BMPs in the bone matrix are released, which induces bone formation [29]. As the matrix breaks down during remodeling, growth factors are released into the surrounding tissue, promoting bone ingrowth. However, when bisphosphonates limit resorption, the release of growth factors is also reduced. This decrease in growth factor release may explain the observed decrease in bone ingrowth distance.

One of the main strengths of our study is the comparative analysis of bone allograft harvested using the Marburg system, in combination with zolendronic acid, during both the early and late stages of bone defect healing. This method enabled us to observe a positive effect on allograft resorption but a negative impact on bone formation.

However, our study has some limitations. The direct effect of zolendronic acid on bone tissue and the appropriate dosage for local use remains controversial. Further research is needed to investigate how zolendronic acid affects cells involved in osteogenesis and to determine the optimal local dosage of zolendronic acid that can inhibit resorption while enhancing the reparative regeneration of bone tissue. Addressing these research questions is essential for improving our understanding of the clinical use of zolendronic acid in bone tissue engineering. Further studies are needed to explain the effect of bisphosphonates on heat-treated bone allografts in humans in a clinical setting. In addition, investigating the impact of bisphosphonates on osteoblasts and osteocytes during bone regeneration and remodeling in vivo is also an area that requires further research.

## Conclusion

We confirmed that the local administration of zolendronic acid with heat-treated bone allograft had negative effects on bone formation in a modified rabbit model of bone defect. Nevertheless, our study showed several limitations and requires further research. While some studies have reported positive effects of zolendronic acid, our study suggests that the optimal dose and concentration of the drug may need further investigation. Specifically, our results suggest that the dose of zolendronic acid used in our study may have been too high and could have contributed to the observed negative impact on bone formation. Additionally, future studies should aim to elucidate the underlying mechanisms of the effects of ZA and explore the potential influence of other osteoinductive factors in the combined use.

## Data Availability

The datasets generated and/or analyzed during the current study are available from the corresponding author upon reasonable request.
